# Anaphylaxis associated with exercise

**DOI:** 10.1186/2045-7022-1-S1-S14

**Published:** 2011-08-12

**Authors:** George Du Toit

**Affiliations:** 1 St Thomas' Hospital, London, UK

## 

Exercise-induced Anaphylaxis (EIA) is a rare, unpredictable, potentially fatal, syndrome characterised by anaphylaxis associated with exercise. Anaphylaxis is associated with a mortality rate of between 1-2%; importantly, some 5-15% of anaphylactic episodes are caused by /or are associated with exercise. EIA may occur independently of food allergen ingestion, or may require the ingestion of a food allergen around the time of exercise, known as Food-dependent exercise-induced anaphylaxis (FDEIA). Concomitant medication use may also be required as an additional facilitating factor, known as ‘summation anaphylaxis'. There are now more than one hundred reviews on the topic of EIA (food dependent and non-food dependent) upon which much of our current knowledge of the condition is based. The aim of this presentation is to draw on key clinical features of these conditions; we will then either support, or challenge, existing hypotheses with respect to exercise-induced pathophysiological mechanisms that may underlie EIA. This will be in the context of recent advances in our understanding of exercise physiology.

